# Automated Assessment of Upper Extremity Function with the Modified Mallet Score Using Single-Plane Smartphone Videos

**DOI:** 10.3390/s25051619

**Published:** 2025-03-06

**Authors:** Cancan Su, Lianne Brandt, Guangwen Sun, Kaitlynn Sampel, Edward D. Lemaire, Kevin Cheung, Albert Tu, Natalie Baddour

**Affiliations:** 1 Children’s Hospital of Eastern Ontario Research Institute, Ottawa, ON K1H 8L1, Canada; gsu@cheo.on.ca (C.S.); lbrandt@cheo.on.ca (L.B.); ksun@cheo.on.ca (G.S.); ksamp081@uottawa.ca (K.S.); 2 Department of Mechanical Engineering, University of Ottawa, Ottawa, ON K1N 6N5, Canada; 3 Faculty of Medicine, University of Ottawa, Ottawa, ON K1H 8M2, Canada; elemaire@uottawa.ca; 4 Department of Surgery, Division of Plastic Surgery, Children’s Hospital of Eastern Ontario, Ottawa, ON K1H 8L1, Canada; kcheung@cheo.on.ca; 5 Department of Surgery, University of Ottawa, Ottawa, ON K1N 6N5, Canada; atu@cheo.on.ca; 6 Department of Surgery, Division of Neurosurgery, Children’s Hospital of Eastern Ontario, Ottawa, ON K1H 8L1, Canada

**Keywords:** modified Mallet score, OpenPose, pose estimation, two-dimensional coordinates, smartphone, video, clinically relevant interpretation

## Abstract

The Modified Mallet Score (MMS) is widely used to assess upper limb function but requires evaluation by experienced clinicians. This study automated MMS assessments using smartphone videos, artificial intelligence (AI), and new algorithms. A total of 125 videos covering all MMS grades were recorded from four neurotypical participants. For all recordings, an expert physician provided manual scores as the ground truth. The OpenPose BODY25 model extracted body keypoint data, which were used to calculate joint angles for an automated scoring algorithm. The algorithm’s scores were compared to the ground truth and expert manual scoring. High accuracy was achieved for the global abduction, hand-to-neck, hand-on-spine, and hand-to-mouth movements, with Pearson correlation coefficients (PCCs) > 0.9 and a low root mean square error (RMSE). Although slightly less accurate for global external rotation, the algorithm still showed strong agreement. This study demonstrates the potential of using AI and smartphone videos for reliable, remote upper limb assessments.

## 1. Introduction

Upper extremity functional assessments guide clinical decision making and help determine healthcare interventions [1,2]. Various assessment tools are available to analyze and objectively quantify upper extremity movement, including scales which visually score range of motion and functional abilities [2,3]. Despite having demonstrated utility, these traditional assessment methods are laborious and time-consuming [1,4,5]. Furthermore, evaluations traditionally require in-person interaction with an experienced clinician. Given Canada’s unique geography and centralization of healthcare resources, access to such specialists is variable and can be impeded by numerous factors, including distance, ability to travel, and regional availability of expertise. With the help of artificial intelligence (AI), virtual upper extremity analysis (VUEA) is a potential substitute that could permit the automated evaluation of specific functions from videos captured with commercially available smartphones.

The Modified Mallet Score (MMS) [6,7] is a clinically validated scoring system for evaluating upper extremity function in children with brachial plexus injuries [3,7]. While many agree that the MMS is a dependable tool [8] and the current standard of care, it is susceptible to inter- and intra-rater variability [5,9,10] and requires interpretation by an experienced clinician and, typically, in-person assessment [5]. Moreover, the results can be affected by complex compensatory movements made by the patient [11]. Grip et al. [12] used inertial sensors to improve the MMS clinical assessment process, with moderate-to-excellent inter-rater reliability and high validity. Unfortunately, these improvements were offset by challenges of acquiring, maintaining, and applying the hardware, as well as additional sensor data which must be collected and subsequently analyzed and interpreted.

To address traditional MMS limitations, a marker-less video analysis approach was developed, automating MMS. This new approach uses AI-based video analysis, where joint and body segment keypoints are identified directly from video footage of the person performing MMS movements [13,14,15,16]. This approach does not require specialized hardware since accessible smartphone video recordings can be used to capture patient movements. Since videos can be captured in any video-friendly areas, MMS evaluation accessibility is improved, especially for people who benefit from remote assessments. A successful automation algorithm has the potential to improve reliability since a consistent set of rules are used to generate MMSs, which also facilitates MMS use when experienced scorers are not available to administer the test.

This research investigated the accuracy of a new automated MMS approach, where MMS movement videos captured using smartphone cameras were processed with the OpenPose pose detection model [17,18,19], and new automated scoring algorithms were applied to calculate MMS grades. By applying an AI-augmented analysis pipeline to smartphone video data, our methods allow for assessments to be completed remotely, which not only improves access to MMS analysis and reduces clinician burden, but could also be used in clinics where videos can be captured and analyzed before specialist appointments, thereby making the patient encounter more efficient.

## 2. Methods

### 2.1. Smartphone Video Recordings

To assess whether the new algorithm provided accurate grading for each MMS category, four volunteer neurotypical adult participants without movement deficits were recorded using a commercially available smartphone while they demonstrated each MMS movement across all possible grades. Two participants were male and two were female. The participants were between 18 and 45 years of age.

A total of 125 videos were captured, grouped into 5 sets of 25 videos, where each set included all 25 possible MMS grades (i.e., 5 grades × 5 MMS movements). To achieve five sets from four participants, one male participant completed two MMS sets. The participants were provided with documents outlining the descriptions, demonstrations, and scoring criteria for each MMS movement to ensure correct understanding and prevent potential biases. This approach ensured that videos for each possible MMS scoring outcome were included in the dataset, which may not occur with a clinical dataset. All 125 videos were evaluated by a physician experienced in MMS evaluation (plastic and reconstructive surgeon who specializes in upper limb trauma) to confirm that all possible MMS grades were appropriately represented across the video sets. Any observed deviations were corrected by re-recording the videos to align with the intended score. Thus, grades assigned to each video were defined as the **ground truth scores**.

Videos were collected using a Samsung Galaxy S23 with (Samsung Electronics Co., Ltd., Suwon, Republic of Korea) a 6.1 display and a 50 MP resolution rear-facing camera under ambient lighting. The smartphone was held manually, without a fixed tripod, to validate the intended implementation (i.e., community setting). The camera operator held the smartphone steadily in front of the participant, ensuring that the participant’s head, hands, and buttocks remained within the frame throughout the recording and that the participant occupied at least a quarter of the entire frame. Only the participant was visible in the frame, and no other reflections, objects, or shadows were present. Videos were trimmed (start and end) to remove unessential frames in preparation for analysis and then uploaded to a secure cloud-based storage for further data processing and analysis.

### 2.2. MMS Grades

Three trained reviewers independently provided MMS grades for all the videos. Before evaluating the videos, each reviewer was informed about the criterion for each movement and the scoring standards. The videos were reviewed in a random order. Each reviewer evaluated all the videos independently, blinded to the other reviewers’ scores. These were defined as the **manual scores**.

To create an automated MMS scoring system which used smartphone videos, body segment orientations and movements first needed to be extracted from the video, without human intervention. To achieve this, OpenPose BODY25 [17,18,19] (v1.7.0) was selected as the pose estimation model for extracting joint keypoints from the video frames. An algorithm was then developed in Python to extract relevant information from the identified OpenPose joint keypoints and score the features from the five MMS movements. The scores produced by the algorithm were defined as the **algorithm scores**.

### 2.3. Algorithm Development

**Figure 1:**
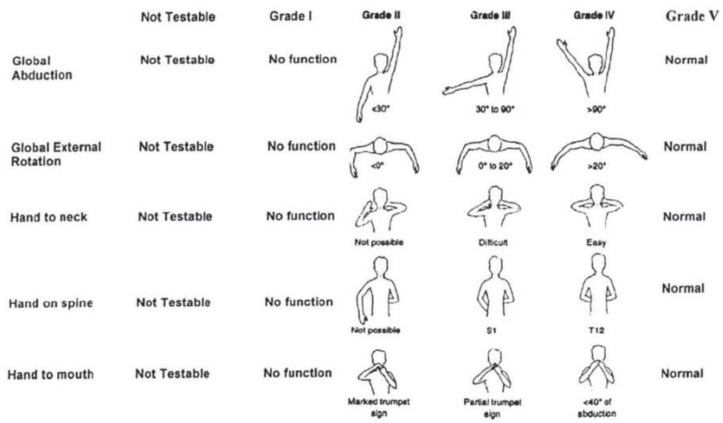
Modified Mallet Scale (modified reproduced figure) [28]. Grade I = no function; Grade V = normal.

The MMS consisted of five movements: global abduction, global external rotation, hand to neck, hand on spine, and hand to mouth. The movements scores ranged from 1 to 5, with 1 being the most affected (no function) and 5 being normal. Figure 1 shows the five MMS movements across all grades. Employing the MMS requires the qualitative assessment of three-dimensional movements, which can pose challenges for interpretation via a single-plane computer vision approach [20,21]. To adapt the MMS for use with conventional two-dimensional (planar) smartphone videos, while also ensuring convenience and accuracy, the following adjustments were implemented:

1. Using trained clinician’s expertise, text descriptions of the different MMS grades were quantified. Parameters and thresholds were established to convert descriptive MMS grades into numerical criteria. Table 1 outlines the ranges of angles (i.e., criterion) when developing a preliminary quantitative interpretation of the MMS grades, for each of the five MMS movements. The criterion (angle) calculations are detailed in Section 2.3.
2. Based on clinician expertise of real-world MMS application, a minimal threshold was defined for determining if a visually quantifiable reduction in mobility would be classified as the same or a different grade. This approach was specifically relevant between Grades IV and V, whereby the movement was scored as Grade V (normal and symmetric to other arm) if the estimated angles provided for each side of the body (i.e., left versus right arm) differed by no more than 15 degrees [22].
3. Considering the capabilities of a single smartphone recording device, as well as the intended recording environment, global external rotation was evaluated from a frontal view (an overhead view would be preferrable but difficult to capture in the community) [23,24].
4. To reduce the chance of occluded joints and body parts [25,26,27], the video recording position for hand on spine was changed from frontal to rear-facing; thus, participant hands remained visible throughout all video frames.

Following this series of modifications, our MMS implementation retained the feasibility and functionality of the traditional MMS while eliminating potential sources of error and better aligning with the goal of data collection in the community.

**Table 1:** Algorithmic Modified Mallet Score sheet. For Grade V, both side of the body are symmetric.

	Global Abduction	Global External Rotation	Hand to Neck	Hand on Spine	Hand to Mouth
**Criterion**	Upper arm angle $(\theta_{UA}$)	$\mathrm{Lower}\;\mathrm{arm}\;\mathrm{transverse}-\mathrm{plane}\;\mathrm{angle}\;(\theta_{LATP}$)	Upper arm angle $(\theta_{UA}$)	Elbow angle $(\theta_{E}$)	Upper arm angle $(\theta_{UA}$)
**Equation**	(1)	(6)	(1)	(7)	(1)
**Grade I**	θ ≤ 15°	θ < −40°	θ < 30°	θ > 155°	Not able to touch
**Grade II**	15° < θ ≤ 30°	−40° < θ ≤ 0°	30° < θ ≤ 50°	115° < θ ≤ 155°	θ > 75°
**Grade III**	30° < θ ≤ 90°	0 < θ ≤ 20°	50° < θ ≤ 80°	95° < θ ≤ 115°	45° < θ ≤ 75°
**Grade IV**	90° < θ ≤ 150°	20° < θ ≤ 40°	80° < θ ≤ 110°	70° < θ ≤ 95°	30° < θ ≤ 45°
**Grade V**	θ > 150°	θ > 40°	θ > 110°	θ < 70°	θ < 30°

**Figure 2:**
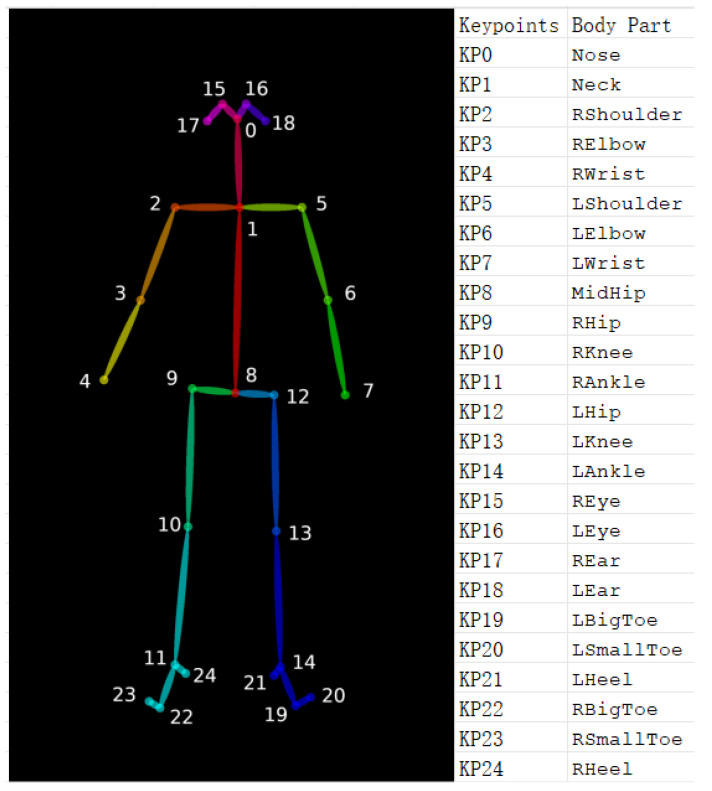
OpenPose BODY-25 model and keypoint legend [16].

**Figure 3:**
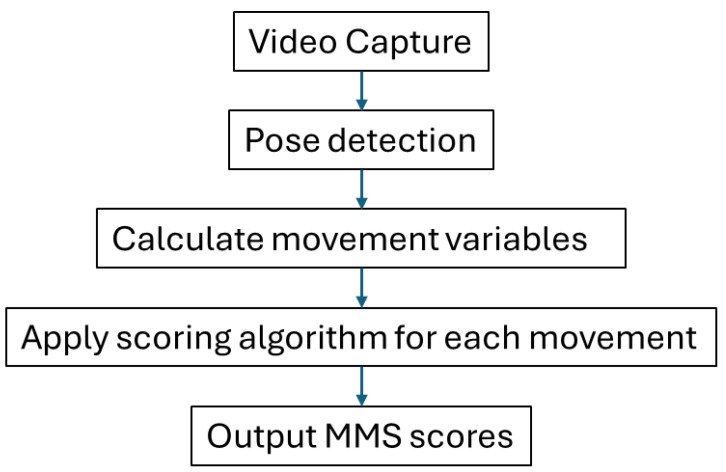
MMS scoring system flowchart.

The 25 keypoints identified by the OpenPose BODY25 model are displayed in Figure 2. OpenPose provides 2D (x, y) keypoint coordinates and confidence scores for each frame in a video. A 5th-order Savitzky–Golay filter [29] with a window size of 13 was employed to smooth all 2D keypoint data before analysis. The angles shown in Table 1 were used by the algorithm to grade each video. A flowchart demonstrating the video processing pipeline can be found in Figure 3.

Specific joint keypoints were used to define vectors representative of body segments, which could be applied in subsequent angle calculations. The general format representing the vector between two keypoints was ${\overset{⃑}{v}}_{first\;keypoint\_second\;keypoint}$. For example, a vector representing the left forearm would be defined using the left elbow (KP6) and left wrist (KP7) keypoints, represented as ${\overset{⃑}{v}}_{elbow\_wrist}$.

As participants performed certain movements, such as moving their arms upwards and over their heads, they could also move their torso/trunk to accommodate for potential mobility issues [30,31]. To mitigate potential errors from participants’ trunk movement, torso direction (${\overset{⃑}{v}}_{t}$) was calculated in each video frame and used as a body position vector from which to calculate the relative arm position. Torso direction (${\overset{⃑}{v}}_{t}$) was calculated as the vector from the neck (KP1) to the hip-midpoint (KP8) (${\overset{⃑}{v}}_{neck\_midhip}$).

#### 2.3.1. Global Abduction

**Figure 4:**
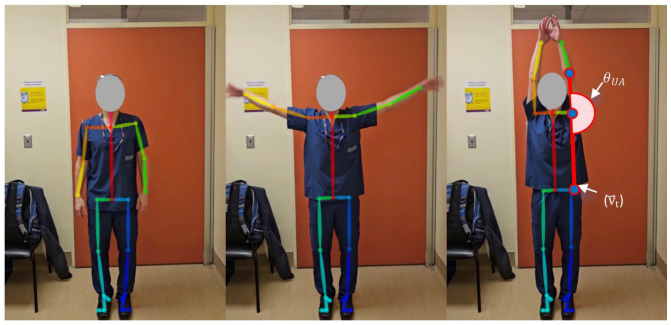
Global abduction.

While standing normally, the participants raised both arms above their head (Figure 4).

The angle formed between a participant’s upper arm and torso direction ($\theta_{UA}$) quantified the global abduction grade. The upper arm vector magnitude (${\overset{⃑}{v}}_{shoulder\_elbow}$), or the distance between the shoulder and elbow of the corresponding arm, was used to calculate the upper arm angle ($\theta_{UA}$) (Equation (1)).

(1) $$\theta_{UA}=arccos\left(\frac{{\overset{⃑}{v}}_{t}\cdot {\overset{⃑}{v}}_{shoulder\_elbow}}{\left||{\overset{⃑}{v}}_{t}||||{\overset{⃑}{v}}_{shoulder\_elbow}|\right|}\right)$$

#### 2.3.2. Global External Rotation

**Figure 5:**
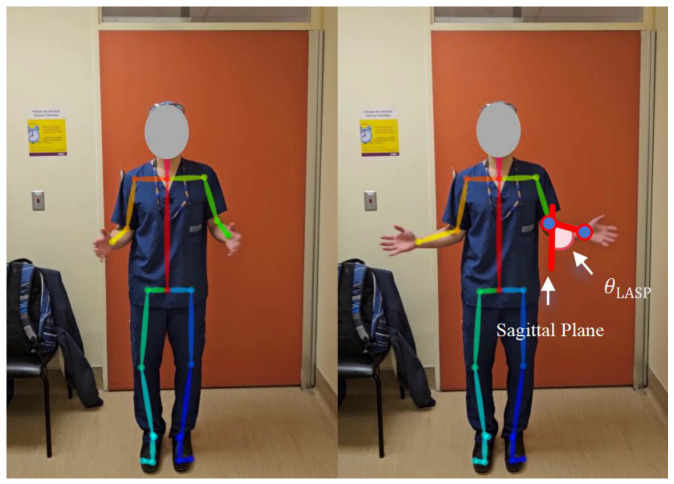
Global external rotation.

The participants were first instructed to stand with their elbows bent at 90°, with their hands in front of their body, forming 90° angles with both arms. Then, the participants rotated their forearms outwards (i.e., away from the body centre) while keeping both elbows flush to their sides/torso and maintaining a 90° angle between the upper and lower arms on both sides (Figure 5).

The angles between the lower arms and the body could be used to quantify a global external rotation grade for each arm (i.e., lower arm transverse-plane angle, $\theta_{LATP}$). When the lower arm was parallel to the sagittal plane (in front of the body), it formed a 0° angle. When the participants moved their arms outwards (moving away from the sagittal plane), the resulting positive angles indicated external rotation. When the participants moved their arms inwards, towards the opposite side of the body, the resulting negative angles indicated internal rotation. Figure 5 shows the movement away from the body.

Some considerations arose when using 2D front-view videos to calculate lower arm transverse-plane angles. A global external rotation is a transverse-plane movement of the forearm, so the motion would be best measured using a 3D or overhead view. Since the 2D videos were taken in the coronal plane, transverse-axis values could not be obtained directly. Therefore, the lower arm transverse-plane angles were based on calculations that used both the length of the projected lower arm vector ($l_{lower\_projected}$) (i.e., lower arm length in the image plane) and the actual lower arm length ($l_{lower}$). The projected lower arm vector was calculated using the following equation:

(2) $$l_{lower\_projected}=||{\overset{⃑}{v}}_{elbow\_wrist}||$$

Length ratios between a participant’s upper and lower arms, for both their right and left arms, were calculated using Equation (3). Since the participant’s arm was in the image plane for the global abduction videos, the global abduction video related to the trial was used to calculate the length ratios between upper ($l_{GAB\_upper})$ and lower ($l_{GAB\_lower})$ arms (i.e., actual length). These lengths are averaged across all frames of the Global Abduction movement video, where n is the total number of frames in the video, to provide an average arm length ratio (${lr}_{GAB})$ that further equates to a Global External Rotation arm length ratio (${lr}_{GER})$, from

(3) $${lr}_{GER}={lr}_{GAB}=\frac{\sum \frac{l_{GAB\_lower}}{l_{GAB\_upper}}}{n}$$

For the global external rotation video, Equation (4) shows how the actual upper arm ($l_{upper})$ length was determined by calculating the vector between the shoulder and the elbow:

(4) $$l_{upper}=||{\overset{⃑}{v}}_{shoulder\_elbow}||$$

With Equation (5), the actual lower arm length ($l_{lower}$) was calculated by the upper arm length ($l_{upper})$ and the arm length ratio (${lr}_{GER}),as\;follows:$

(5) $$l_{lower}=l_{upper}\times {lr}_{GER}$$

Finally, Equation (6) shows how the lower arm transverse-plane angle ($\theta_{LATP}$) calculations incorporated the projected ($l_{lower\_projected}$) and actual ($l_{lower}$) lower arm lengths:

(6) $$\theta_{LATP}=\left\{\begin{array}{c} 90{}^{\circ}-\arccos\left(\frac{l_{lower\_projected}}{l_{lower}}\right)\;\;\;if\;\theta_{LATP}>0{}^{\circ}\\ arccos\left(\frac{l_{lower\_projected}}{l_{lower}}\right)-90{}^{\circ}\;\;\;\;if\;\theta_{LATP}<0{}^{\circ} \end{array}\right.$$

#### 2.3.3. Hand to Neck

**Figure 6:**
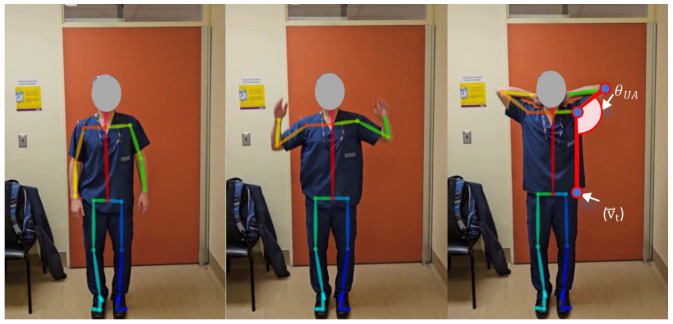
Hand to neck.

The hand-to-neck movement evaluated the ability to simultaneously abduct and externally rotate the shoulder. While standing normally, the participants raised both arms above and behind their head to touch the back of their neck (Figure 6).

The angle formed between the original starting point of each upper arm and the final upper arm location could be used to calculate a hand-to-neck score for each arm (i.e., upper arm angle, $\theta_{UA}$). Calculating the upper arm angle ($\theta_{UA}$) for the hand-to-neck movement followed the same process as global abduction (Equation (1)).

Due to 2D video data constraints, the external rotation measurement was difficult to implement because OpenPose output could not determine whether the hand was behind or in front of the neck or head. Our algorithm calculated an angle for both arms, but if a participant incorrectly positioned one arm in front of their head, it assigned the same MMS grade to both arms. Future work should refine these aspects as pose-estimating algorithms evolve and improve. Current pose estimators do not (accurately) give depth information. If the pose estimator could return accurate depth information, this could be used to help differentiate whether the hands are positioned behind or in front of the head. 

#### 2.3.4. Hand on Spine

**Figure 7:**
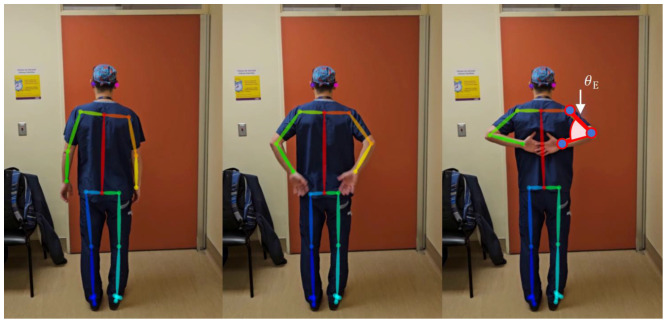
Hand on spine.

The participants were first instructed to turn and face away from the camera. They moved their hands towards the centre of their back and then moved their hands as far up their spine as possible (Figure 7).

The intent of the hand-on-spine movement was to measure where the hand was in relation to the spine, related to the amount of elbow flexion required to move the hand up the back and along the spine. Calculating elbow flexion when a participant’s arms are behind their backs can be used as a surrogate measure for hand-on-spine. The angles formed between the upper and lower arm can then be used to quantify a score for both arms (i.e., elbow angle, $\theta_{E}$). Equation (7) shows how the vector between the elbow and the wrist (${\overset{⃑}{v}}_{elbow\_wrist}$), along with the vector between the elbow and the shoulder (${\overset{⃑}{v}}_{elbow\_shoulder}$), can be used to calculate an elbow angle:

(7) $$\theta_{E}=arccos\left(\frac{{\overset{⃑}{v}}_{elbow\_shoulder}\cdot {\overset{⃑}{v}}_{elbow\_wrist}}{\left||{\overset{⃑}{v}}_{elbow\_shoulder}||||{\overset{⃑}{v}}_{elbow\_wrist}|\right|}\right)$$

#### 2.3.5. Hand to Mouth

**Figure 8:**
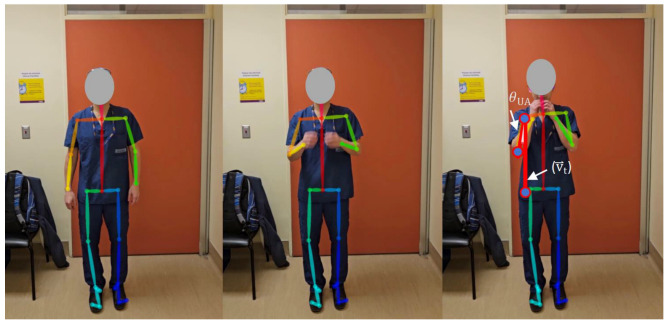
Hand to mouth.

While standing, the participants raised both arms towards their mouth (Figure 8).

The intent of the hand-to-mouth movement was to measure the amount of shoulder abduction that was required to achieve the hand-to-mouth movement. Here, we used the angles formed between the torso direction (${\overset{⃑}{v}}_{t}$) and each upper arm as surrogates to quantify a hand-to-mouth grade for both arms (upper arm angle ($\theta_{UA}$), as shown in Equation (1) and Figure 8).

One consideration in interpreting algorithm-calculated angles for the hand-to-mouth movement is that some videos will capture participants not being able to touch their mouth. As mentioned previously, the algorithm provides interpretations of MMS grades based on comparing the angles calculated from videos against pre-defined criteria. Within the context of the hand-to-mouth movement, this means that it is possible for a participant’s arm to form an angle that would be interpreted as corresponding to a specific grade, while they are not actually touching their mouth. Therefore, an additional consideration for interpretation must be included: if both the distance between the wrist and nose keypoints and the distance between the wrist and shoulder keypoints exceed a certain limit, the movement should be classified as “Not able to touch” or Grade I.

### 2.4. Evaluation Methods

To evaluate automated scoring performance, the Pearson correlation coefficient (PCC) and root mean square error (RMSE) were calculated between (i) the algorithm and ground truth scores, (ii) the manual and ground truth scores, (iii) the automated and manual scores, and (iv) the manual scores.

All videos for each movement were included in the PCC or RMSE calculations (i.e., 25 videos for each of the five movements). However, due to disagreement in scoring between all three reviewers for one hand-to-mouth video, this video was excluded from the performance calculations.

All PCC and RMSE values related to manual scores were calculated by averaging the PCC or RMSE results from each reviewer.

## 3. Results

**Figure 9:**
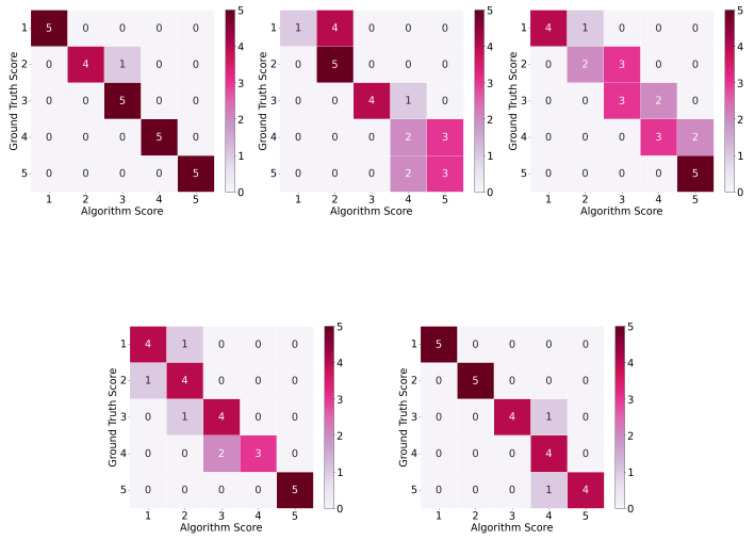
Heatmap for ground truth versus algorithm scores: (**top left**) global abduction, (**top middle**) global external rotation, (**top right**) hand to neck, (**bottom left**) hand on spine, and (**bottom right**) hand to mouth.

Figure 9 displays heatmaps comparing the ground truth and automatic scores for each MMS movement. The heatmaps show that the differences between the automatic and ground truth scores are, at most, within one grade, meaning that any grade misclassifications by the algorithm are within one grade below or above the actual grade of the moment performed in the video.

Table 2 presents the PCC, RMSE, and the respective 95% confidence intervals (95% CIs) between the algorithm and ground truth scores for each MMS movement. Table 3 shows PCC, RMSE and their 95% CIs between the manual scores and the ground truth scores.

The algorithm scores had high correlations with the ground truth scores, greater than 0.94 for all movements except for global external rotation at 0.91. The RMSE values were all less than 0.622 (i.e., less than one scoring category).

The manual versus ground truth results also had high correlations and a low RMSE. The algorithm scores had better results than the manual scores for the global abduction and hand-to-mouth movements. The manual scores had better results for global external rotation. The hand-on-spine and hand-on-neck movements were equivalent between groups.

The PCC, RMSE and their 95% CIs between the algorithm and manual scores are reported in Table 4. Table 5 presents the PCC, RMSE and their 95% CIs between the three manual reviewers.

The algorithm scores had high correlations with the manual scores (PCC: 0.889–0.950), comparable to the correlations between the manual raters (PCC: 0.844–0.975). The RMSE of the algorithm was close to or lower than that of the manual raters for hand-on-spine and hand-to-neck. The RMSE for global external rotation was slightly higher.

Appendix A provides Table A1, Table A2, Table A3, Table A4, Table A5, Table A6, Table A7, Table A8, Table A9 and Table A10 which details correlations and root mean square error values between the scores from the three reviewers, the algorithm, and the ground truth across all five MMS movements.

## 4. Discussion

This research describes a novel method for automatically generating MMS grades from handheld smartphone video data. This algorithmic approach produced viable results that were equivalent or better than manually scored results, indicating that this method could be useful for clinical decision making.

A variety of upper extremity assessment tools are available, but many require multiple steps for both patients and assessors, as well as the use of physical tools or props Despite these limitations, the automation of these other assessment tools could be considered the subject of future research [32,33,34,35].

The participants in this study purposely demonstrated movements that covered the entire range of MMS grades, providing a dataset which allowed for the evaluation of the algorithm’s performance across all MMS grades. Therefore, the outcomes from this study are relevant for people with a broad range of movement capabilities.

In addition, since this approach produced viable results using a handheld smartphone for video collection, automatic MMS scoring could be applied in multiple settings at the point of patient contact.

The algorithm automatically calculated MMS grades that regularly matched the ground truth score or, at most, differed by one score. Correlations to the ground truth scores were above 0.95 for all movements except for global external rotation, with these scores still highly correlated at 0.91. Overall, these results were equivalent to or better than the manually scored videos. The RMSE was also below one for all analyses in this research, showing that the differences in scores were generally below one for both algorithm and manual scoring.

The algorithm’s results correlated well with the average manual score results and produced overall better results than the three reviewers. This is promising, as the algorithmic approach demonstrates comparable performance to manual scoring and aligns closely with the results from trained reviewers.

Global external rotation estimation errors tended to occur when the scores were near the maximum and minimum grades, with four out of five videos scored as Grade I when they should have been Grade II. These errors were likely influenced by the surrogate method for estimating 3D movements from 2D coordinates. As the lower arm approaches the coronal plane (i.e., parallel with the body), the lower arm angle approaches ±90°, and changes in the projected arm length become minimal. This can make it difficult for the algorithm to differentiate between Grades I and II and exacerbates the effect of keypoint estimation errors from OpenPose, leading to a higher likelihood of misjudgments. New pose detection methods that provide an estimated depth axis for each keypoint (i.e., estimate z-axis into the video frame) could be investigated in future research [36].

For the hand-on-neck movement, scoring requires both the ability to lift the arm and externally rotate the shoulder. Quantifying shoulder joint rotation from 2D coordinate data can produce similar errors to global external rotation. In addition, we cannot determine whether the hand is in front of or behind the head from the 2D OpenPose keypoints. Future work to refine the algorithm so as to detect hand occlusion would likely enhance the scoring of this movement, alongside research with new pose detection models which provide a 3D depth coordinate.

A limitation of this study was that only four able-bodied participants were recruited to generate the test dataset. This approach was successful in providing multiple instances of each MMS grades, for all five movements, which allowed for a complete assessment of algorithm scoring across the full range of the MMS. However, the next phase of research should evaluate the new automated MMS algorithm with a sufficient sample size of in-clinic patient videos.

## 5. Conclusions

This research demonstrated that handheld smartphone videos can be used with AI-based pose detection and custom algorithms to automatically evaluate upper extremity function and provide a clinically relevant interpretation using a validated visual scoring system (MMS). This approach appears to be highly reproducible and reliable and has the potential to greatly expand access to upper extremity function assessment.

The next steps will involve testing the algorithm’s performance in patients with upper extremity dysfunction and investigating applications in real-world scenarios. Additionally, new methods to improve the algorithms for hand-on-neck and global external rotation will be investigated. The adoption of AI-driven systems in this domain offers advantages, including increased efficiency, consistent scoring, and scalability. As automated methods advance, healthcare professionals can be supported by reducing manual workload through providing them with tools to support clinical decision-making processes.

## Figures and Tables

**Table 2:** Pearson’s correlation coefficient (PCC), root mean square error (RMSE), and 95% confidence intervals (95% CIs) between algorithm and ground truth scores.

	PCC [95% CI]	RMSE [95% CI]
**Global Abduction**	0.990 [0.978, 0.996]	0.200 [0.120, 0.278]
**Global External Rotation**	0.910 [0.805, 0.960]	0.632 [0.436, 0.828]
**Hand to Neck**	0.945 [0.878, 0.976]	0.566 [0.379, 0.752]
**Hand on Spine**	0.953 [0.895, 0.979]	0.447 [0.287, 0.607]
**Hand to Mouth**	0.981 [0.956, 0.992]	0.283 [0.174, 0.391]

**Table 3:** Average Pearson’s correlation coefficient (PCC), root mean square error (RMSE), and 95% confidence intervals (95% CIs) between manual and ground truth scores.

	PCC [95% CI]	RMSE [95% CI]
**Global Abduction**	0.916 [0.824, 0.962]	0.544 [0.144, 1.056]
**Global External Rotation**	0.987 [0.954, 0.992]	0.210 [0.130, 0.289]
**Hand to Neck**	0.920 [0.826, 0.964]	0.622 [0.324, 0.921]
**Hand on Spine**	0.977 [0.948, 0.990]	0.325 [0.202, 0.448]
**Hand to Mouth**	0.919 [0.823, 0.964]	0.585 [0.236, 0.934]

**Table 4:** Average Pearson’s correlation coefficient (PCC), root mean square error (RMSE), and 95% confidence intervals (95 % CIs) between algorithm and manual scores.

	PCC [95% CI]	RMSE [95% CI]
**Global Abduction**	0.921 [0.834, 0.964]	0.533 [0.143, 1.044]
**Global External Rotation**	0.909 [0.802, 0.960]	0.663 [0.465, 0.861]
**Hand to Neck**	0.889 [0.769, 0.949]	0.786 [0.283, 1.290]
**Hand on Spine**	0.950 [0.889, 0.978]	0.446 [0.287, 0.605]
**Hand to Mouth**	0.910 [0.805, 0.960]	0.610 [0.207, 1.014]

**Table 5:** Average Pearson’s correlation coefficient (PCC), root mean square error (RMSE), and 95% confidence intervals (95 % CIs) between the three manual scorers.

	PCC [95% CI]	RMSE [95% CI]
**Global Abduction**	0.844 [0.685, 0.928]	0.747 [0.058, 1.632]
**Global External Rotation**	0.975 [0.944, 0.989]	0.359 [0.226, 0.492]
**Hand to Neck**	0.865 [0.720, 0.939]	0.860 [0.264, 1.455]
**Hand on Spine**	0.975 [0.944, 0.989]	0.325 [0.202, 0.448]
**Hand to Mouth**	0.854 [0.693, 0.934]	0.763 [0.299, 1.227]

## Data Availability

Data are contained within the article.

## Appendix A

**Table A1:** Correlation between the scores of three reviewers, the algorithm, and the ground truth in global abduction.

	Ground Truth	Algorithm	Reviewer 1	Reviewer 2	Reviewer 3
**Ground Truth**	1.0000	0.9904	0.9701	0.8041	0.9733
**Algorithm**	0.9904	1.0000	0.9812	0.8173	0.9656
**Reviewer 1**	0.9701	0.9812	1.0000	0.7886	0.9812
**Reviewer 2**	0.8041	0.8173	0.7886	1.0000	0.7625
**Reviewer 3**	0.9733	0.9656	0.9812	0.7625	1.0000

**Table A2:** Correlation between the scores of three reviewers, the algorithm, and the ground truth in global external rotation.

	Ground Truth	Algorithm	Reviewer 1	Reviewer 2	Reviewer 3
**Ground Truth**	1.0000	0.9453	0.9662	0.8616	0.9311
**Algorithm**	0.9453	1.0000	0.9660	0.7774	0.9228
**Reviewer 1**	0.9662	0.9660	1.0000	0.8128	0.9603
**Reviewer 2**	0.8616	0.7774	0.8128	1.0000	0.8232
**Reviewer 3**	0.9311	0.9228	0.9603	0.8232	1.0000

**Table A3:** Correlation between the scores of three reviewers, the algorithm, and the ground truth in hand to neck.

	Ground Truth	Algorithm	Reviewer 1	Reviewer 2	Reviewer 3
**Ground Truth**	1.0000	0.9453	0.9662	0.8616	0.9311
**Algorithm**	0.9453	1.0000	0.9660	0.7774	0.9228
**Reviewer 1**	0.9662	0.9660	1.0000	0.8128	0.9603
**Reviewer 2**	0.8616	0.7774	0.8128	1.0000	0.8232
**Reviewer 3**	0.9311	0.9228	0.9603	0.8232	1.0000

**Table A4:** Correlation between the scores of three reviewers, the algorithm, and the ground truth in hand on spine.

	Ground Truth	Algorithm	Reviewer 1	Reviewer 2	Reviewer 3
**Ground Truth**	1.0000	0.9531	0.9746	0.9817	0.9746
**Algorithm**	0.9531	1.0000	0.9415	0.9479	0.9613
**Reviewer 1**	0.9746	0.9415	1.0000	0.9723	0.9810
**Reviewer 2**	0.9817	0.9479	0.9723	1.0000	0.9723
**Reviewer 3**	0.9746	0.9613	0.9810	0.9723	1.0000

**Table A5:** Correlation between the scores of three reviewers, the algorithm, and the ground truth in hand to mouth.

	Ground Truth	Algorithm	Reviewer 1	Reviewer 2	Reviewer 3
**Ground Truth**	1.0000	0.9808	0.9032	0.9479	0.9060
**Algorithm**	0.9808	1.0000	0.9006	0.9263	0.9042
**Reviewer 1**	0.9032	0.9006	1.0000	0.8574	0.8807
**Reviewer 2**	0.9479	0.9263	0.8574	1.0000	0.8238
**Reviewer 3**	0.9060	0.9042	0.8807	0.8238	1.0000

**Table A6:** RMSE between the scores of three reviewers, the algorithm, and the ground truth in global abduction.

	Ground Truth	Algorithm	Reviewer 1	Reviewer 2	Reviewer 3
**Ground Truth**	0.0000	0.2000	0.3464	0.9381	0.3464
**Algorithm**	0.2000	0.0000	0.2828	0.9165	0.4000
**Reviewer 1**	0.3464	0.2828	0.0000	0.9592	0.2828
**Reviewer 2**	0.9381	0.9165	0.9592	0.0000	1.0000
**Reviewer 3**	0.3464	0.4000	0.2828	1.0000	0.0000

**Table A7:** RMSE between the scores of three reviewers, the algorithm, and the ground truth in global external rotation.

	Ground Truth	Algorithm	Reviewer 1	Reviewer 2	Reviewer 3
**Ground Truth**	0.0000	0.6325	0.3464	0.2828	0.0000
**Algorithm**	0.6325	0.0000	0.6633	0.6928	0.6325
**Reviewer 1**	0.3464	0.6633	0.0000	0.4472	0.3464
**Reviewer 2**	0.2828	0.6928	0.4472	0.0000	0.2828
**Reviewer 3**	0.0000	0.6325	0.3464	0.2828	0.0000

**Table A8:** RMSE between the scores of three reviewers, the algorithm, and the ground truth in hand to neck.

	Ground Truth	Algorithm	Reviewer 1	Reviewer 2	Reviewer 3
**Ground Truth**	0.0000	0.5657	0.4000	0.9381	0.5292
**Algorithm**	0.5657	0.0000	0.4000	1.2961	0.6633
**Reviewer 1**	0.4000	0.4000	0.0000	1.1314	0.4472
**Reviewer 2**	0.9381	1.2961	1.1314	0.0000	1.0000
**Reviewer 3**	0.5292	0.6633	0.4472	1.0000	0.0000

**Table A9:** RMSE between the scores of three reviewers, the algorithm, and the ground truth in hand on spine.

	Ground Truth	Algorithm	Reviewer 1	Reviewer 2	Reviewer 3
**Ground Truth**	0.0000	0.4472	0.3464	0.2828	0.3464
**Algorithm**	0.4472	0.0000	0.4899	0.4472	0.4000
**Reviewer 1**	0.3464	0.4899	0.0000	0.3464	0.2828
**Reviewer 2**	0.2828	0.4472	0.3464	0.0000	0.3464
**Reviewer 3**	0.3464	0.4000	0.2828	0.3464	0.0000

**Table A10:** RMSE between the scores of three reviewers, the algorithm, and the ground truth in hand to mouth.

	Ground Truth	Algorithm	Reviewer 1	Reviewer 2	Reviewer 3
**Ground Truth**	0.0000	0.2828	0.6325	0.4899	0.6325
**Algorithm**	0.2828	0.0000	0.6325	0.5657	0.6325
**Reviewer 1**	0.6325	0.6325	0.0000	0.7483	0.6928
**Reviewer 2**	0.4899	0.5657	0.7483	0.0000	0.8485
**Reviewer 3**	0.6325	0.6325	0.6928	0.8485	0.0000

## References

[1] Francisco-Martínez C., Prado-Olivarez J., Padilla-Medina J.A., Díaz-Carmona J., Pérez-Pinal F.J., Barranco-Gutiérrez A.I., Martínez-Nolasco J.J. (2021). Upper Limb Movement Measurement Systems for Cerebral Palsy: A Systematic Literature Review. Sensors.

[2] Wagner L.V., Davids J.R. (2012). Assessment tools and classification systems used for the upper extremity in children with cerebral palsy. Clin. Orthop. Relat. Res..

[3] Gupta S., Srinivasan N., Mahajan J., Song A., Chu A., McGrath A. (2022). Outcome measures in OBPP. Brachial Plex. Inj. New Tech. Ideas.

[4] Bourke-Taylor H. (2003). Melbourne Assessment of Unilateral Upper Limb Function: Construct validity and correlation with the Pediatric Evaluation of Disability Inventory. Dev. Med. Child. Neurol..

[5] van der Sluijs J.A., van Doorn-Loogman M.H., Ritt M.J., Wuisman P.I. (2006). Interobserver reliability of the Mallet score. J. Pediatr. Orthop. B.

[6] Al-Qattan M.M., El-Sayed A.A. (2014). Obstetric brachial plexus palsy: The mallet grading system for shoulder function-revisited. Biomed. Res. Int..

[7] Sibiński M., Synder M. (2010). Soft tissue rebalancing procedures with and without internal rotation osteotomy for shoulder deformity in children with persistent obstetric brachial plexus palsy. Arch. Orthop. Trauma. Surg..

[8] Nath R.K., Karicherla P., Mahmooduddin F. (2010). Shoulder function and anatomy in complete obstetric brachial plexus palsy: Long-term improvement after triangle tilt surgery. Childs Nerv. Syst..

[9] Delioğlu K., Unes S., Tuncdemir M., Ozal C., Bıyık K.S., Uzumcugil A. (2024). Interrater reliability of face-to-face, tele- and video-based assessments with the modified Mallet classification in brachial plexus birth injuries. J. Hand Surg. Eur. Vol..

[10] Russo S.A., Chafetz R.S., Rodriguez L.M., Roposh C.M., Zlotolow D.A., Kozin S.H., Gaughan J.P., Richards J.G. (2022). Comparison of Shoulder Motion Measurements by Visual Estimate, Goniometer and Motion Capture. J. Pediatr. Orthop..

[11] Lovette M., Chafetz R.S., Russo S.A., Kozin S.H., Zlotolow D.A. (2024). Shoulder Motion Overestimated by Mallet Scores. J. Pediatr. Orthop..

[12] Grip H., Källströmer A., Öhberg F. (2022). Validity and Reliability of Wearable Motion Sensors for Clinical Assessment of Shoulder Function in Brachial Plexus Birth Injury. Sensors.

[13] Hill S.W., Mong S., Vo Q. (2022). Three-dimensional motion analysis for occupational therapy upper extremity assessment and rehabilitation: A scoping review. Open J. Occup. Ther..

[14] Dev T., Reetajanetsureka, Selvaraj S., Magimairaj H.P., Balasubramanian S. (2022). Accuracy of Single RGBD Camera-Based Upper-Limb Movement Tracking Using OpenPose. Converging Clinical and Engineering Research on Neurorehabilitation, I.V. Proceedings of the 5th International Conference on Neurorehabilitation (ICNR2020), Online, 13–16 October 2020.

[15] Debnath B., O’brien M., Yamaguchi M., Behera A. (2022). A review of computer vision-based approaches for physical rehabilitation and assessment. Multimed. Syst..

[16] Albuquerque P., Machado J.P., Verlekar T.T., Correia P.L., Soares L.D. (2021). Remote Gait Type Classification System Using Markerless 2D Video. Diagnostics.

[17] Ramesh S.H., Lemaire E.D., Cheung K., Tu A., Baddour N. (2023). Automated Stride Detection from OpenPose Keypoints Using Handheld Smartphone Video. Proceedings of the 2023 IEEE Sensors Applications Symposium (SAS).

[18] Zhang F., Juneau P., McGuirk C., Tu A., Cheung K., Baddour N., Lemaire E. (2021). Comparison of OpenPose and HyperPose artificial intelligence models for analysis of hand-held smartphone videos. Proceedings of the 2021 IEEE International Symposium on Medical Measurements and Applications (MeMeA).

[19] Ramesh S.H., Lemaire E.D., Tu A., Cheung K., Baddour N. (2023). Automated Implementation of the Edinburgh Visual Gait Score (EVGS) Using OpenPose and Handheld Smartphone Video. Sensors.

[20] Gold S., Rangarajan A., Lu C.-P., Pappu S., Mjolsness E. (1998). New algorithms for 2D and 3D point matching: Pose estimation and correspondence. Pattern Recognit..

[21] Konrad J., Wang M., Ishwar P. (2012). 2d-to-3d image conversion by learning depth from examples. Proceedings of the 2012 IEEE Computer Society Conference on Computer Vision and Pattern Recognition Workshops.

[22] Parkinson A.O., Apps C.L., Morris J.G., Barnett C.T., Lewis M.G.C. (2021). The Calculation, Thresholds and Reporting of Inter-Limb Strength Asymmetry: A Systematic Review. J. Sports Sci. Med..

[23] Hsu C.-C.J., Lu M.-C., Lu Y.-Y. (2011). Distance and angle measurement of objects on an oblique plane based on pixel number variation of CCD images. IEEE Trans. Instrum. Meas..

[24] Elassal N., Elder J.H. (2017). Estimating Camera Tilt from Motion without Tracking. Proceedings of the 2017 14th Conference on Computer and Robot Vision (CRV).

[25] Dursun A.A., Tuncer T.E. (2021). Estimation of partially occluded 2D human joints with a Bayesian approach. Digit. Signal Process..

[26] Rafi U., Gall J., Leibe B. A semantic occlusion model for human pose estimation from a single depth image. Proceedings of the IEEE Conference on Computer Vision and Pattern Recognition Workshops.

[27] Zhang T., Huang B., Wang Y. Object-occluded human shape and pose estimation from a single color image. Proceedings of the IEEE/CVF Conference on Computer Vision and Pattern Recognition.

[28] Mahon J., Malone A., Kiernan D., Meldrum D. (2015). Three dimensional movement analysis of the upper limb during activities of daily living in children with obstetric brachial plexus palsy: Comparison with healthy controls. Gait Posture.

[29] Schafer R.W. (2011). What is a savitzky-golay filter? [lecture notes]. IEEE Signal Process. Mag..

[30] Peeters L.H., de Groot I.J., Geurts A.C. (2018). Trunk involvement in performing upper extremity activities while seated in neurological patients with a flaccid trunk–A review. Gait Posture.

[31] Levin M.F., Liebermann D.G., Parmet Y., Berman S. (2016). Compensatory versus noncompensatory shoulder movements used for reaching in stroke. Neurorehabilit. Neural Repair.

[32] Huang J., Ji J.R., Liang C., Zhang Y.Z., Sun H.C., Yan Y.H., Xing X.B. (2022). Effects of physical therapy-based rehabilitation on recovery of upper limb motor function after stroke in adults: A systematic review and meta-analysis of randomized controlled trials. Ann. Palliat. Med..

[33] Moiziard V., Lansaman T., Mauruc Soubirac E., Revol M., Coulet B., Hugeron C., Gelis A., Laffont I. (2022). Assessment of the upper limb of the tetraplegic patient. Hand Surg. Rehabil..

[34] Velstra I.M., Ballert C.S., Cieza A. (2011). A systematic literature review of outcome measures for upper extremity function using the international classification of functioning, disability, and health as reference. PMR.

[35] Proud E.L., Miller K.J., Bilney B., Balachandran S., McGinley J.L., Morris M.E. (2015). Evaluation of measures of upper limb functioning and disability in people with Parkinson disease: A systematic review. Arch. Phys. Med. Rehabil..

[36] Dill S., Rösch A., Rohr M., Güney G., De Witte L., Schwartz E., Hoog Antink C. (2023). Accuracy Evaluation of 3D Pose Estimation with MediaPipe Pose for Physical Exercises. Curr. Dir. Biomed. Eng..

